# Metagenomic insights into microbial diversity, xenobiotic and plastic-degrading enzymes in sediments of river Yamuna at Agra

**DOI:** 10.3389/fmicb.2026.1828736

**Published:** 2026-06-03

**Authors:** Ajaya Kumar Rout, Partha Sarathi Tripathy, Sushree Swati Rout, Neelesh Kumar, Satya Narayan Parida, Anusuiya Panda, Deepa Suman, Anuj Tyagi, Bijay Kumar Behera, Pramod Kumar Pandey

**Affiliations:** 1 College of Fisheries (Datia), Rani Lakshmi Bai Central Agricultural University, Jhansi, Uttar Pradesh, India; 2 ICAR-Central Inland Fisheries Research Institute, Kolkata, West Bengal, India; 3 Department of Zoology, Fakir Mohan University, Balasore, Odisha, India; 4 College of Agriculture, Rani Lakshmi Bai Central Agricultural University, Jhansi, Uttar Pradesh, India; 5 National Fisheries Development Board, Department of Fisheries, Government of India, Hyderabad, Telangana, India

**Keywords:** metagenomics, microbial diversity, plastic degrading enzyme, river Yamuna, sediment, xenobiotic degradation

## Abstract

The river Yamuna is one of the most polluted rivers in India and is heavily impacted by urban, industrial, and agricultural inputs. In this study, shotgun metagenomics was used to investigate microbial diversity and functional potential in sediments from three locations: Balkeshwar Shivpuri Agra (BSA), Taj Ganj Yamuna (TGY), and Yamuna Expressway Agra (YEA). A total of 38.3–46.5 million reads per sample were generated, yielding 3.08–5.54 million predicted ORFs. Taxonomic profiling revealed that BSA exhibited higher microbial diversity, with a more even distribution of dominant taxa compared to TGY and YEA. Functional analysis indicated that core metabolic pathways (e.g., glycolysis and TCA cycle) were more abundant in BSA, whereas pathways related to aromatic compound degradation were relatively enriched in TGY. Plastic-degrading enzyme homologs were detected across all sites, with the strongest signals associated with biodegradable polymers such as polyhydroxyalkanoates (PHA), polyhydroxybutyrate (PHB), and polyethylene glycol (PEG). The normalized abundances of PHA-associated enzymes were approximately 60–75% higher in YEA compared to BSA and TGY, while homologs linked to recalcitrant plastics such as polyethylene (PE) and low-density polyethylene (LDPE) were detected at low levels across all sites (< 10–15%). Similarly, xenobiotic degradation pathways, including chlorocyclohexane and chlorobenzene degradation, showed relatively higher abundance in YEA, whereas bisphenol degradation was more prominent in BSA. Overall, the results indicate that sediment microbial communities along the river Yamuna harbor diverse metabolic capabilities and functional potential for pollutant degradation, with site-specific variations driven by local environmental conditions.

## Introduction

The river Yamuna, the largest tributary of the Ganga, holds immense mythological and historical significance in India. Originating from the Yamunotri Glacier in the lower Himalayas, the river traverses Uttarakhand, Himachal Pradesh, Haryana, Delhi, and Uttar Pradesh before merging with the Ganga at Prayagraj. Major urban centers such as Delhi, Agra, Mathura, and Prayagraj are located along its banks, relying on the river for diverse domestic, agricultural, and industrial needs. However, the Yamuna now faces severe pollution arising primarily from the untreated discharge of urban runoff, including fecal and hospital waste, domestic sewage, and industrial effluents, which introduces excessive organic loads, toxic compounds, and heavy metals into the river system (Mittal et al., 2019; Samson et al., 2020). Highly polluted stretches, notably in Delhi (e.g., Okhla, Wazirabad, Faizpur) as well as various Ghats in Agra, have exhibited alarming levels of chemical and microbial contamination (Isaac and Siddiqui, 2022; Parida et al., 2022). This pollution is further compounded by the escalating impacts of agricultural practices and insufficient waste treatment, which collectively exacerbate the environmental degradation of the river Yamuna.

Agricultural intensification in the Yamuna catchment contributes significantly to pollution through the use and runoff of chemical fertilizers and pesticides, further increasing toxicant accumulation in riverine waters and sediments (Anand et al., 2019; Kumar et al., 2020). The situation is exacerbated by insufficient municipal treatment infrastructure, resulting in continuous input of untreated municipal and industrial waste and a consequent decline in water quality and ecological function (Isaac and Siddiqui, 2022). This has led to elevated biological oxygen demand (BOD), frequently exceeding permissible thresholds, and a marked reduction in dissolved oxygen, severely impacting aquatic biota (Raghav et al., 2024). These ongoing pollution problems underscore the urgency of addressing the combined effects of agricultural runoff and inadequate waste management, which contribute to the river's declining health and its adverse impacts on human and ecological wellbeing.

The deteriorating water quality of the Yamuna poses serious health risks for both local populations and aquatic organisms. Studies have reported associations between exposure to contaminants in the river (including heavy metals, persistent organic pollutants, and emerging contaminants such as pharmaceuticals and personal care products) and a spectrum of human health concerns, including carcinogenic and non-carcinogenic outcomes, endocrine disruption, and increased incidences of waterborne diseases (Parween et al., 2021; Babuji et al., 2023). Persistent environmental pollution also threatens riverine biodiversity and the sustainability of ecosystem services that support millions of people throughout northern India (Khan et al., 2021). This ongoing contamination, driven by urban and industrial waste, not only exacerbates health risks but also accelerates the decline in water quality, further stressing the river's ecosystems and the communities dependent on its resources.

The river Yamuna is severely impacted by multiple anthropogenic pollution sources, primarily including untreated domestic sewage, industrial effluents, and agricultural runoff. Rapid urbanization and population growth have resulted in large volumes of municipal wastewater entering the river, often without adequate treatment, while industrial discharges contribute toxic chemicals and heavy metals (Mittal et al., 2019; Isaac and Siddiqui, 2022). In addition, agricultural practices introduce fertilizers and pesticides that further degrade water quality (Anand et al., 2019; Kumar et al., 2020). These combined inputs significantly increase organic load, nutrient enrichment, and contaminant accumulation in both water and sediments, ultimately impairing ecological health and ecosystem functioning (Parida et al., 2022; Raghav et al., 2024).

Microorganisms play a pivotal role in maintaining and regulating ecosystem processes, particularly within riverine environments. River sediments provide unique, heterogeneous habitats that support highly diverse microbial assemblages, which in turn drive essential ecosystem functions. These sediment-based microbial communities are actively involved in numerous metabolic pathways, responsible for nutrient cycling, regulation of biogeochemical and biophysical processes, and facilitation of energy flow within river ecosystems (Mittal et al., 2019). Sediments typically harbor greater microbial diversity than overlying waters, acting as reservoirs for taxa equipped with diverse metabolic capabilities (Rathour et al., 2020). Changes in terms of abundance, diversity, or functional attributes of sediment microbial communities in response to environmental gradients or anthropogenic disturbances serve as sensitive indicators of river ecosystem health and quality (Rajeev et al., 2021). Monitoring these communities provides valuable insight into ecosystem resilience, potential biogeochemical shifts, and the overall status of riverine environments. This intricate relationship between microorganisms and sediment ecosystems highlights the importance of monitoring microbial communities in river sediments, as they not only serve as indicators of environmental health but also play a critical role in nutrient and pollutant cycling, particularly in areas impacted by human activity.

Microorganisms, inhabiting river sediments, are key drivers of metabolic activities that underpin biogeochemical cycling and energy flow in river ecosystems (Ibekwe et al., 2016). River sites, impacted by anthropogenic activities, often serve as reservoirs for pathogenic microorganisms and fecal indicator bacteria, posing significant public health risks. Industrial effluents, sewage contamination, agricultural runoff, and inadequate waste management practices introduce a range of pathogens and contaminants into these aquatic systems (Rout et al., 2022, 2024). Consequently, characterizing the microbiome within polluted sediment environments can aid in identifying bioindicator species and microbial communities responsive to specific pollutants. Such biomarkers are pivotal for assessing the ecological function and health status of river ecosystems (Qiu et al., 2020). This shift toward metagenomic analysis is transforming our understanding of microbial ecosystems in river sediments, offering unprecedented insights into the diversity, function, and resilience of microbial communities, and highlighting their potential role in bioremediation and pollutant management (Rout et al., 2025).

Understanding the complexity and interrelationships of microbial communities in aquatic ecosystems remains challenging when relying solely on traditional culture-based techniques. Whole-genome metagenomic analysis, leveraging next-generation sequencing (NGS) technologies, has emerged as a critical tool for elucidating the taxonomic diversity of uncultivable microorganisms and uncovering key microbial metabolic pathways involved in pollutant bioremediation (Reddy and Dubey, 2019; Samson et al., 2020). Recent scientific advances have overcome many limitations of culture-dependent methods, facilitating comprehensive phylogenetic and functional genomic profiling of environmental microbiomes (Rout et al., 2024). Moreover, integrated metagenomics and bioinformatics approaches have enabled the identification of beneficial microbial taxa from river sediments with potential industrial and biotechnological applications. Our ongoing research has identified numerous advantageous and bioremediative bacterial species in metagenomic datasets, derived from the sediments of rivers Ganga and Yamuna (Behera et al., 2020a,b). Metagenomic analyses of the Yamuna sediment have also detected diverse antimicrobial resistance gene (ARG) classes (Das et al., 2020). Complementary studies by various research groups continue to elucidate the structure, diversity, and functional attributes of microbial communities inhabiting river sediments through metagenomic methodologies (Abia et al., 2018; Chen et al., 2019; Mittal et al., 2019; Reddy and Dubey, 2019).

This study represents the first comprehensive whole-genome metagenomic analysis of sediment samples from three key sites along the river Yamuna in Agra, Balkeshwar Shivpuri (BSA), Taj Ganj Yamuna (TGY), and Yamuna Expressway Agra (YEA). Focusing on bacterial diversity and functional potential, the investigation employed advanced bioinformatics to identify genes encoding xenobiotic and plastic-degrading enzymes in metagenomes. Given the pivotal ecological roles that microorganisms play in nutrient cycling and environmental homeostasis, understanding the microbial composition and their potential pollutant-degrading capabilities is critically essential for polluted river systems such as Yamuna, which receives significant domestic, industrial, and miscellaneous contaminants. The present study, therefore, aimed to unravel the diverse bacterial community structure and to functionally characterize their metabolic activities, using a shotgun metagenomics approach. In addition, the relationships between microbiome composition, functional gene content, and the physicochemical properties of water and sediment were systematically assessed. The findings offer in-depth insight into the bacterial diversity, cataloging the gene and enzyme repertoire as well as metabolic pathways active in highly polluted stretches of the Yamuna. Notably, novel microbial taxa, plastic and xenobiotic-degrading enzymes were discovered at all three locations, advancing our understanding of the river's ecological health and its intrinsic bioremediation potential.

## Methods

### Study area and sample collection

**Figure 1:**
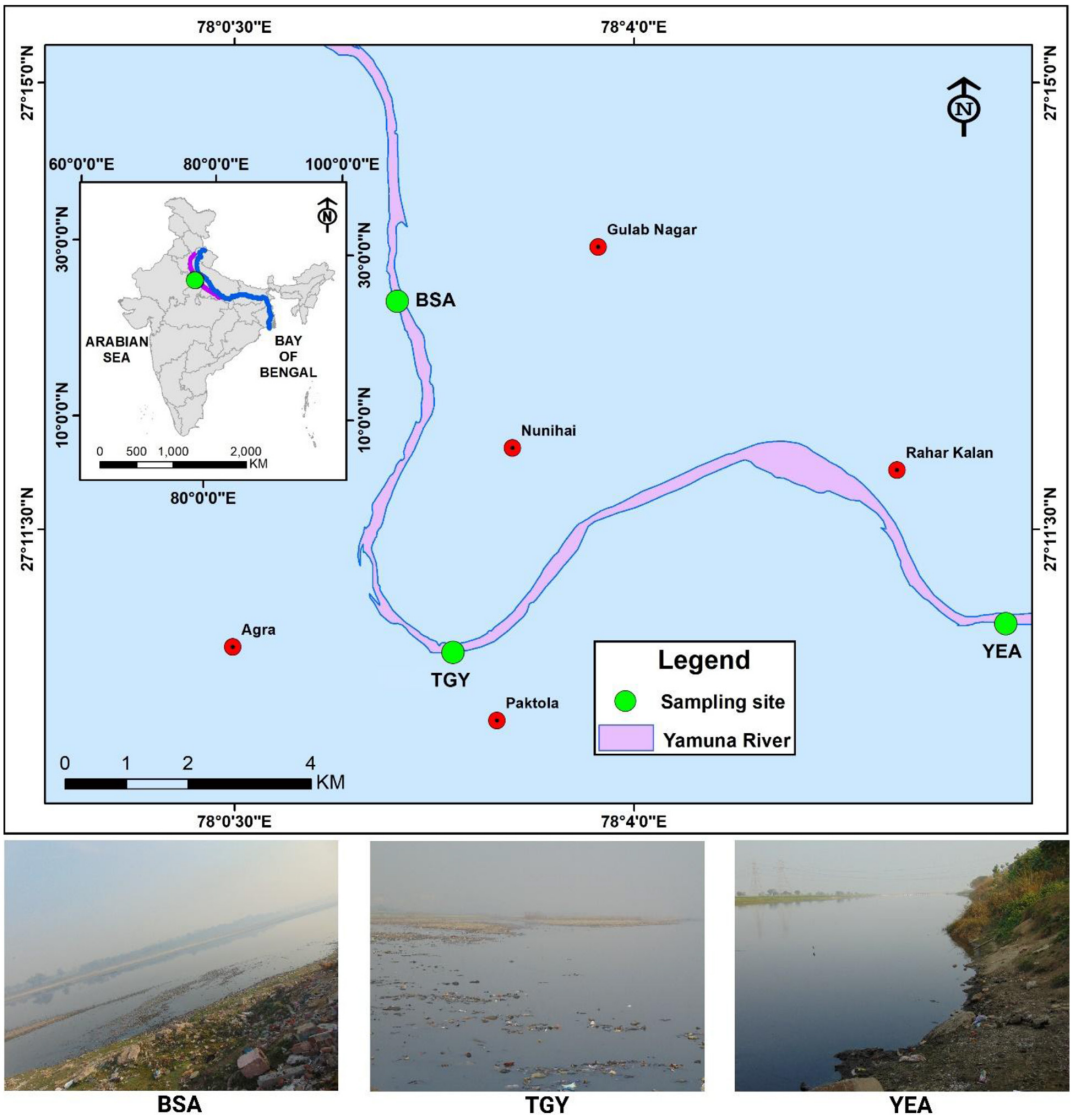
GIS map depicts the locations of the sampling sites situated at Agra stretch in river Yamuna, i.e., Balkeshwar Shivpuri Agra (BSA), Taj Ganj Yamuna (TGY), and Yamuna Expressway Agra (YEA) near Agra, Uttar Pradesh, India. The map of sediment sampling areas was constructed in ArcGIS 10.2.1 platform.

Sediment and water samples were collected from three sites along the river Yamuna near Agra, Uttar Pradesh, India: Balkeshwar Shivpuri Agra (27.221461 N, 78.03204 E), Taj Ganj Yamuna (27.175609 N, 78.040207 E), and Yamuna Expressway Agra (27.179351 N, 78.120992 E; Figure 1). Sampling occurred between 08:30 and 10:30 a.m. in March 2021. At each site, composite samples were prepared by pooling five sediment subsamples (~250 m intervals; total ~500 g wet weight per site) and collecting 500 mL of water, stored separately in autoclaved amber glass bottles labeled by site i.e., Balkeshwar Shivpuri Agra (BSA), Taj Ganj Yamuna (TGY), Yamuna Expressway Agra (YEA). On-site measurements of pH (Hanna Instruments, Sigma, USA) and temperature (MT-222 Digiflexi, Dr. Morepen, India) were recorded. The sediment samples were collected in sterile plastic bags, sealed, and shipped at 4 °C before being stored at −80 °C to facilitate further study. At each sampling location, five sediment subsamples collected along a ~250 m stretch from a depth of 10–20 cm were pooled to generate a single composite sample, which was used for DNA extraction and sequencing (Xiong et al., 2025). Thus, the metagenomic analysis represents one composite biological sample per site rather than independent biological replicates. Sequencing was performed on a single library per site, without technical replication, although quality control measures were applied during library preparation and sequencing to ensure data reliability. The use of composite sampling was intended to capture spatial heterogeneity within each site while providing a representative overview of the microbial community. The approximate inter-site distances are approximately 25–30 km.

However, it is important to note that hydrological variables such as flow velocity, discharge, sediment transport, and deposition dynamics were not explicitly measured or incorporated in the sampling design. Riverine sediment layers, particularly at depths of 10–20 cm, may represent a mixture of recently deposited and reworked materials influenced by resuspension and transport processes (Kleeberg et al., 2013). Therefore, the collected sediment may not necessarily reflect a fully stabilized microbial community but could represent a transitional assemblage undergoing adaptation to fluctuating environmental conditions.

The three sampling locations i.e., BSA, TGY and YEA were selected to represent distinct environmental conditions and anthropogenic influences along the Agra stretch of the river Yamuna. BSA is influenced by urban and domestic inputs and represents a comparatively mixed-use zone with moderate pollution levels. TGY is located near densely populated and tourist-impacted areas, receiving substantial sewage discharge and exhibiting elevated organic and chemical loads. In contrast, YEA is situated downstream in a relatively less disturbed stretch but receives cumulative upstream inputs, representing a zone of pollutant accumulation and altered physicochemical conditions.

### Analysis of physicochemical parameters

Physicochemical analyses were performed on sediment and water samples from all three river Yamuna sites. Water quality parameters, including dissolved oxygen (DO), total dissolved solids (TDS), pH, biological oxygen demand (BOD), chemical oxygen demand (COD), salinity, and specific conductivity (μS/cm), were determined following standard APHA protocols. Sediment characteristics, including total nitrogen (%), organic carbon (OC), pH, and available phosphate, were also assessed in accordance with APHA guidelines (Eaton et al., 1995). Elemental composition of sediments was quantified using inductively coupled plasma mass spectrometry (ICP-MS).

### Genomic DNA isolation, library preparation and sequencing

Metagenomic DNA was extracted from sediment samples using the XpressDNA Soil Kit (MagGenome, USA) following the manufacturer's protocol which includes inhibitor removal steps for humic substances. The DNA samples' quality and quantity were determined using a 1% agarose gel electrophoresis and purity was evaluated using a Nanodrop™ (Thermo Scientific, USA), respectively, and stored at a temperature of −80 °C until further use. A quality standard for constructing the DNA fragment library was defined as an optical density (OD) absorbance value of 1.8–2.0 at a 260/280 purity ratio, with a DNA quantity of ≥ 1 μg. DNA concentration was further quantified using a fluorometric method i.e., Qubit dsDNA HS Assay (Thermo Fisher Scientific, USA) to ensure accurate quantification prior to library preparation.

Purified DNA was sent to Genotypic Technology Pvt. Ltd. (Bangalore, India) for library preparation and sequencing. Paired-end libraries were constructed using the NEBNext Ultra DNA Library Prep Kit (New England Biolabs, USA) per manufacturer instructions. Genomic DNA was fragmented using sonication, followed by end repair, adapter ligation and PCR enrichment. Library purification was performed using the MinElute PCR Purification Kit (Qiagen, UK). Library size distribution was assessed using agarose gel electrophoresis against HyperLadder IV (Bioline, UK). Equimolar pooled libraries underwent 2 × 150 bp paired-end sequencing on the Illumina HiSeq 2500 platform (rapid run mode), with duplicate samples distributed across two lanes.

### Data submission to SRA

Metagenomic sequencing data have been deposited in the NCBI Sequence Read Archive (SRA) under accession numbers SRR16085952 (BSA), SRR16085951 (TGY), and SRR16085950 (YEA).

### Sequence analysis and taxonomic classification

The quality of raw reads was checked on fastqc v0.12.0. Then, the raw reads were quality trimmed via cutadapt v5.2 followed by assembly preparation using MEGAHIT v1.2.9. The assembly generated via MEGAHIT was annotated using Prokka v1.15.6. Raw paired-end FASTQ reads from all three samples were uploaded to the MG-RAST v4.0.3 server (Keegan et al., 2016). Pre-processing included adapter trimming and quality filtering, with removal of artificially replicated reads using DRISEE. The Bowtie tool (Langmead and Salzberg, 2012) was used to filter out near-exact matched sequences from Homo sapiens. rDNA sequences were identified via vsearch against SSU, Greengenes, and RDP databases (97% identity threshold). Reads were clustered at 97% identity using cd-hit, and representative sequences were annotated against these databases using BLAT (min. identity 97%, e-value 1e-06, min. alignment 50 bp). The SSU database yielded the highest hit coverage and was selected for downstream analyses.

Functional annotation and KEGG pathway analysis were performed using MG-RAST. RMA files generated in MEGAN6 (Beier et al., 2017) were visualized with the Interactive Tree of Life (iTOL) tool to identify reads associated with xenobiotic degradation pathways. Taxonomic profiling was conducted using Kraken2 v2.1.6 (Wood et al., 2019) with Bracken v3.1 (Lu et al., 2017) abundance estimation. Kraken-Bracken-plot.py (https://github.com/rotheconrad/Kraken-Bracken-plot) generated species-level abundance plots (top 50 taxa), and pavian v1.0 (Breitwieser and Salzberg, 2020) was used for interactive visualization of overall microbial community composition across samples. Sequences labeled as “uncultured bacterium” represent reads or ORFs that could not be confidently assigned to a known taxon in the reference database.

### Prediction of plastic degrading enzymes

Predicted protein-coding sequences (ORFs) obtained from assembled contigs using Prokka v1.15.6 were assigned a taxonomic label using Kraken2 v2.1.6 with Bracken v3.1 abundance estimation, based on exact k-mer matches to the reference database. To identify plastic-degrading functions, all predicted protein sequences were queried against the PlasticDB database using BLASTP with an e-value threshold of ≤ 1e-6, minimum sequence identity ≥70%, and minimum alignment coverage ≥70%. For each ORF with a significant hit to PlasticDB, the corresponding taxonomic assignment (from Kraken2/Bracken) was used to link the functional annotation (plastic-degrading enzyme) to a taxon within the same metagenomic dataset. Species-plastic combinations were quantified by location, and diversity patterns were visualized using ggplot2 in R (Wickham, 2011). To enable robust cross-site comparisons and account for differences in sequencing depth, assembly size, and predicted gene content, functional annotations related to plastic-degrading bacteria were normalized prior to analysis. Specifically, counts of species–plastic associations were expressed as ORF-normalized abundance, obtained by dividing counts by the total number of predicted open reading frames (ORFs) for each site. The normalization approach minimizes biases arising from unequal sequencing output and gene prediction across samples (BSA, TGY, and YEA), thereby allowing biologically meaningful comparisons.

### Statistical data analysis

Metagenomic data processing and analysis were conducted using multiple computational pipelines to characterize microbial diversity and plastic/xenobiotic degradation potential across the three river Yamuna sites (BSA, TGY, YEA). Raw reads were pre-processed for quality control (adapter trimming, duplicate removal) using MG-RAST and DRISEE. Taxonomic profiling employed Kraken2/Bracken with abundance visualization of top 50 species per site, comparative community analysis via Pavian, and phylogenetic trees constructed at the species level. Functional annotation via KEGG pathway analysis (MG-RAST/MEGAN6) identified xenobiotic degradation pathways, while PlasticDB queries quantified the potential for plastic-degrading enzymes. Bacterial-plastic degradation diversity was visualized using ggplot2 in R. Statistical comparisons were used to assess site-specific differences in microbial composition and degradation capacity. As each sampling site was represented by a composite sample rather than independent biological replicates, statistical analyses were conducted across functional categories rather than replicate samples. Therefore, *p*-values should be interpreted as indicative of distributional differences among features rather than strict inferential comparisons between independent biological replicates.

## Results

### Physicochemical analysis of sediment and water samples

Water and sediment samples were obtained from different sites of river Yamuna, viz, BSA, TGY, and YEA near Agra, Uttar Pradesh, India (Figure 1). The water quality parameters exhibited elevated levels of BOD (4.2–23.9 ppm) and COD (13–32.9 ppm) recorded at Agra, Uttar Pradesh locations (BSA, TGY, YEA; Table S1). The sediment quality characteristics indicated that Agra exhibited higher levels of Organic carbon (ranging from 0.12 to 0.48%; Table 1). The physico-chemical indicators, associated with pollution, exhibited elevated levels compared to the sampling sites in Agra. Based on the analysis of physicochemical parameters, it can be concluded that Agra exhibited higher levels of pollution (Table 1). The DO was very low in the YEA sample and high in the BSA sample in the river Yamuna (Table S1). The water quality parameters have been compiled and shown in Table S1.

**Table 1:** Physicochemical parameters and heavy metals of sediment at different sampling sites.

Parameters	BSA	TGY	YEA
pH	8.1	7.3	7.56
Specific conductivity (μS/cm)	282	363	383
Organic carbon (%)	0.35	0.12	0.48
Available phosphate (mg/100 g)	0.38	1.75	0.89
Total nitrogen (%)	0.05	0.05	0.06
Manganese (μg/g) (dry weight basis)	199.2	135.9	132.1
Iron (μg/g) (dry weight basis)	8,512.6	10,234.4	8,441.1
Copper (μg/g) (dry weight basis)	14.1	117.9	26.9
Zinc (μg/g) (dry weight basis)	29.0	193.7	48.8
Cadmium (μg/g) (dry weight basis)	0.1	13.8	1.6
Lead (μg/g) (dry weight basis)	2.7	7.4	23.1
Chromium (μg/g) (dry weight basis)	25.8	49.4	25.4

### Sample information and descriptive sequence statistics

The raw read and assembly statistics for the three samples (BSA, TGY, and YEA) are summarized as follows. For BSA, a total of 38,343,727 sequences with a length of 5,751,559,050 bp were obtained, with 93.50% of reads having a quality score >Q20. The assembly generated 2,731,848 contigs, of which 50,538 were shorter than 150 bp, and 2,681,310 were longer than 150 bp. The predicted ORFs totaled 3,083,326, with 2,186,940 being ≥150 bp. In TGY, 43,593,647 sequences with a total length of 6,539,047,050 bp were sequenced, achieving 96.50% of reads with a quality score >Q20. The assembly resulted in 4,357,687 contigs, with 82,548 shorter than 150 bp and 4,275,139 longer than 150 bp. The predicted ORFs amounted to 4,821,175, with 3,380,076 ≥150 bp. Lastly, for YEA, 46,540,984 sequences were obtained, totaling 6,981,147,600 bp, with 95.50% of reads having a quality score >Q20. The assembly yielded 4,960,271 contigs, of which 85,995 were shorter than 150 bp, and 4,874,276 were longer than 150 bp. The predicted ORFs totaled 5,543,201, with 3,883,324 being ≥150 bp. Detailed data is provided in Table 2.

**Table 2:** Sequencing and assembly statistics were conducted on three sediment samples collected from the river Yamuna at Agra, Uttar Pradesh.

Location	Raw read statistics			Assembly statistics				
	Total no. of sequences	Total length of sequence in bp	% of reads >Q20	Total No. of Contigs	Contigs of length<150 bp	Contigs of length >150 bp	Total predicted ORFs	Total no. of ORFs with minimum length 150 bp
BSA	38,343,727	5,751,559,050	93.50	2,731,848	50,538	2,681,310	3,083,326	2,186,940
TGY	43,593,647	6,539,047,050	96.50	4,357,687	82,548	4,275,139	4,821,175	3,380,076
YEA	46,540,984	6s,981,147,600	95.50	4,960,271	85,995	4,874,276	5,543,201	3,883,324

### Taxonomic abundance of microbial taxa

**Figure 2:**
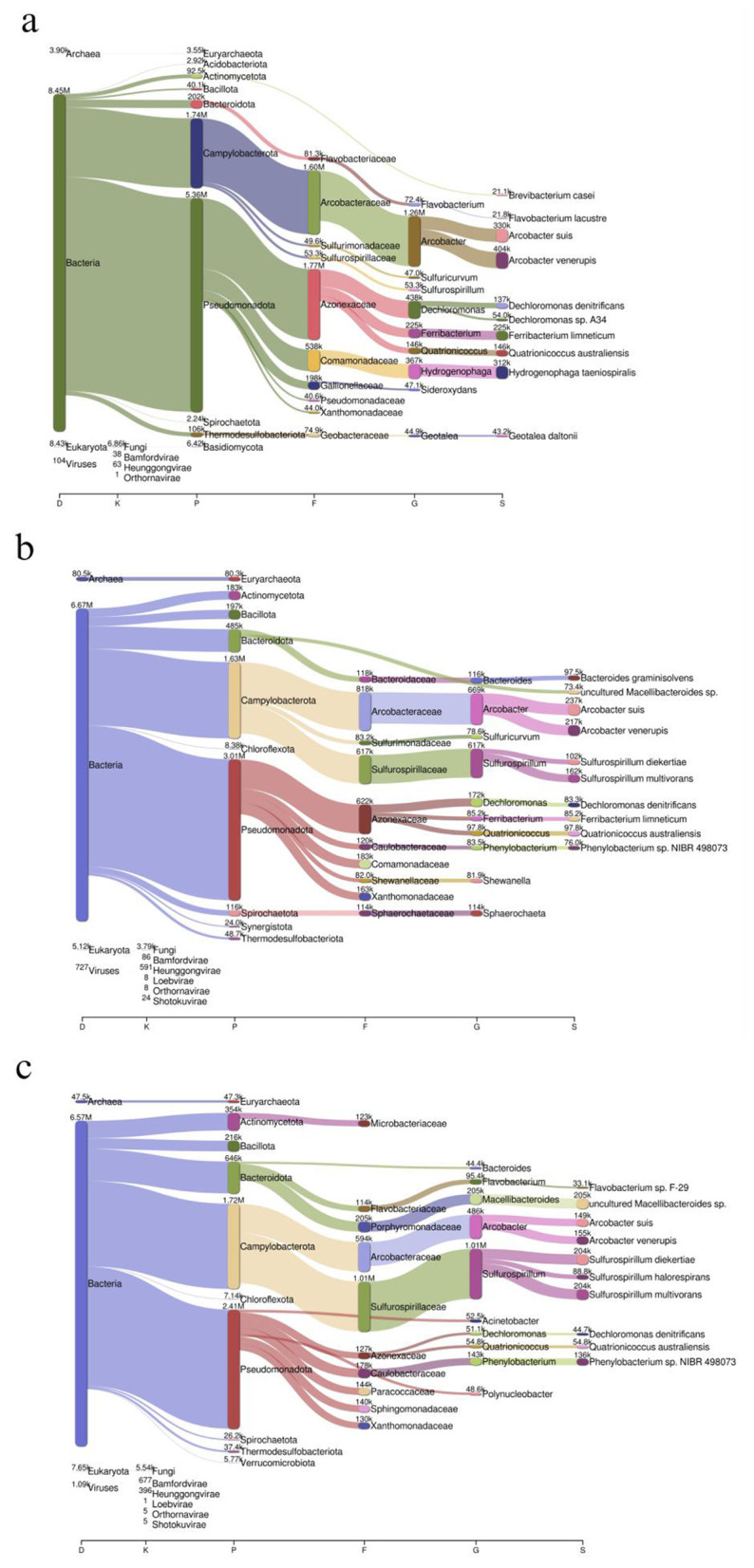
Sankey plot illustrating the distribution and abundance of microbial species across three locations in river Yamuna: **(a)** BSA, **(b)** TGY, and **(c)** YEA. Each plot represents the flow of microbial species from the sampled environment to their respective taxonomic categories, with the thickness of the flow indicating the relative abundance of each species. The colored bands represent distinct taxonomic groups, and the microbial species are listed on the right, showing the distribution across the different locations.

**Figure 3:**
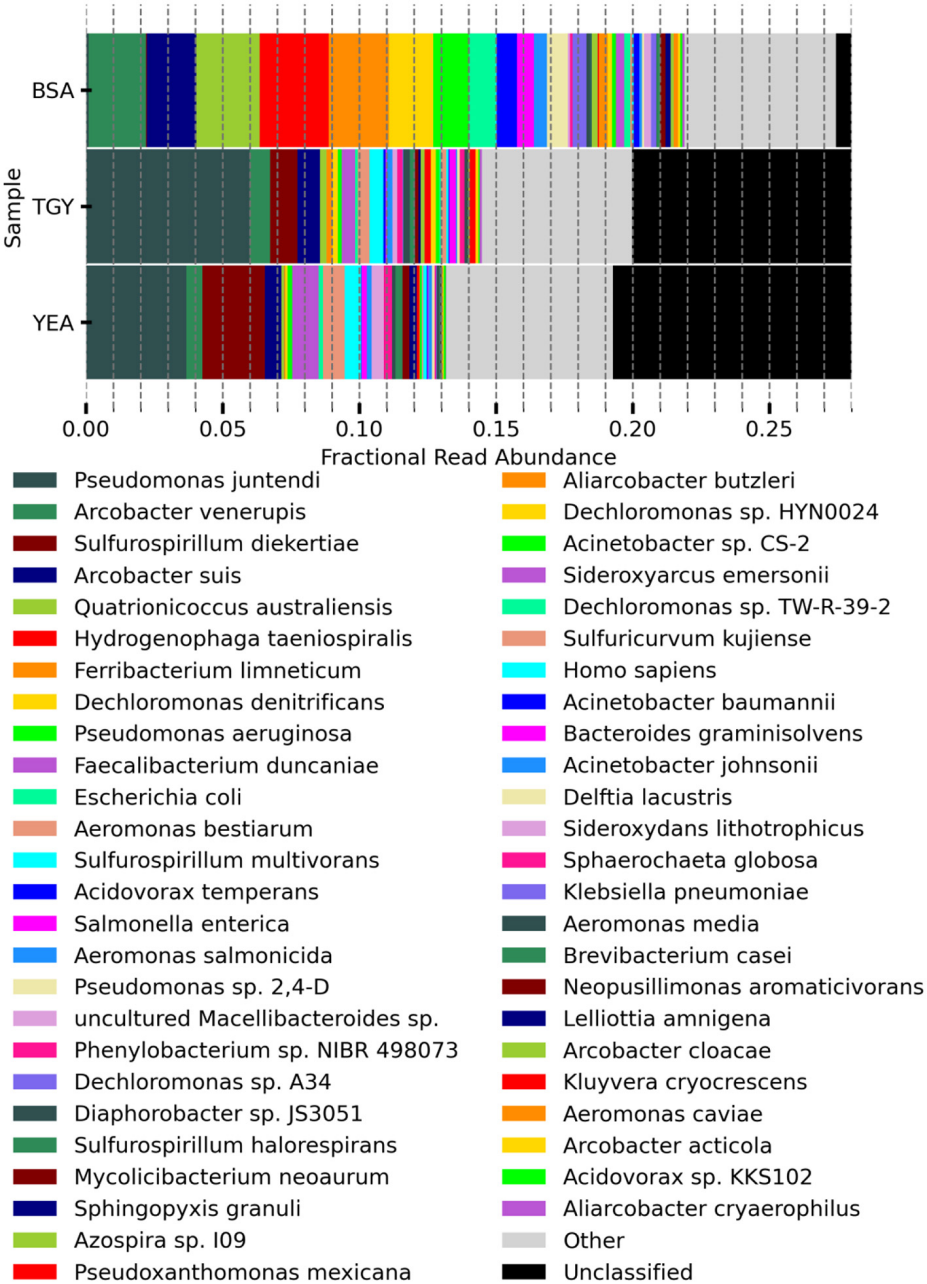
Taxonomic diversity plot displaying the top 50 species identified in three locations along the river Yamuna (BSA, TGY, YEA) based on Kraken-Bracken analysis. The bar graph represents the fractional read abundance for each species across the three locations, with colors indicating different species as shown in the legend. Each sample is labeled by location, and the *x*-axis corresponds to the fractional read abundance of the species. The plot was generated using Kraken-Bracken-plot.py (https://github.com/rotheconrad/Kraken-Bracken-plot), which highlights the diversity and relative abundance of microbial communities at the sites.

The taxonomic abundance analysis of different microbial species across the three locations, using both pavian (Figure 2) and Kraken-Bracken-plot.py (Figure 3), showed species diversity along the river Yamuna. At each sampling location, species abundance was quantified by fractional read abundance, which indicated the proportion of each species relative to the total species pool within a sample. The graphical representation shows distinct patterns in microbial community distribution across the three locations.

BSA exhibited a diverse and fairly balanced distribution of microbial species. The analysis indicates the presence of a wide range of species, with particular dominance by *Pseudomonas junendi* and *Arcobacter venerupis*. Other notable species at BSA include *Sulfurospirillum diekteriae, Hydrogenophaga taeniospiralis*, and *Pseudomonas aeruginosa*. TGY showed a slightly less diverse distribution than BSA, with some species highly abundant. The most prominent observed species were *Acinetobacter* sp. *CS-2* and *Aliarcobacter butzleri*. Interestingly, some species, such as *Dechloromonas* sp. *HYN0024* and *Sideroxyarcus emersonii* also showed consistent presence, with higher fractional read abundances across multiple samples. YEA showed a significantly different microbial composition, with several species showing low fractional read abundance. Species such as *Aliarcobacter butzleri, Acinetobacter baumannii*, and *Dechloromonas denitrificans* were observed, but their fractional read abundances were lower compared to the other locations. Notably, the species *Pseudomonas aeruginosa* was prominently present in the YEA area.

### KEGG pathway analysis

**Figure 4:**
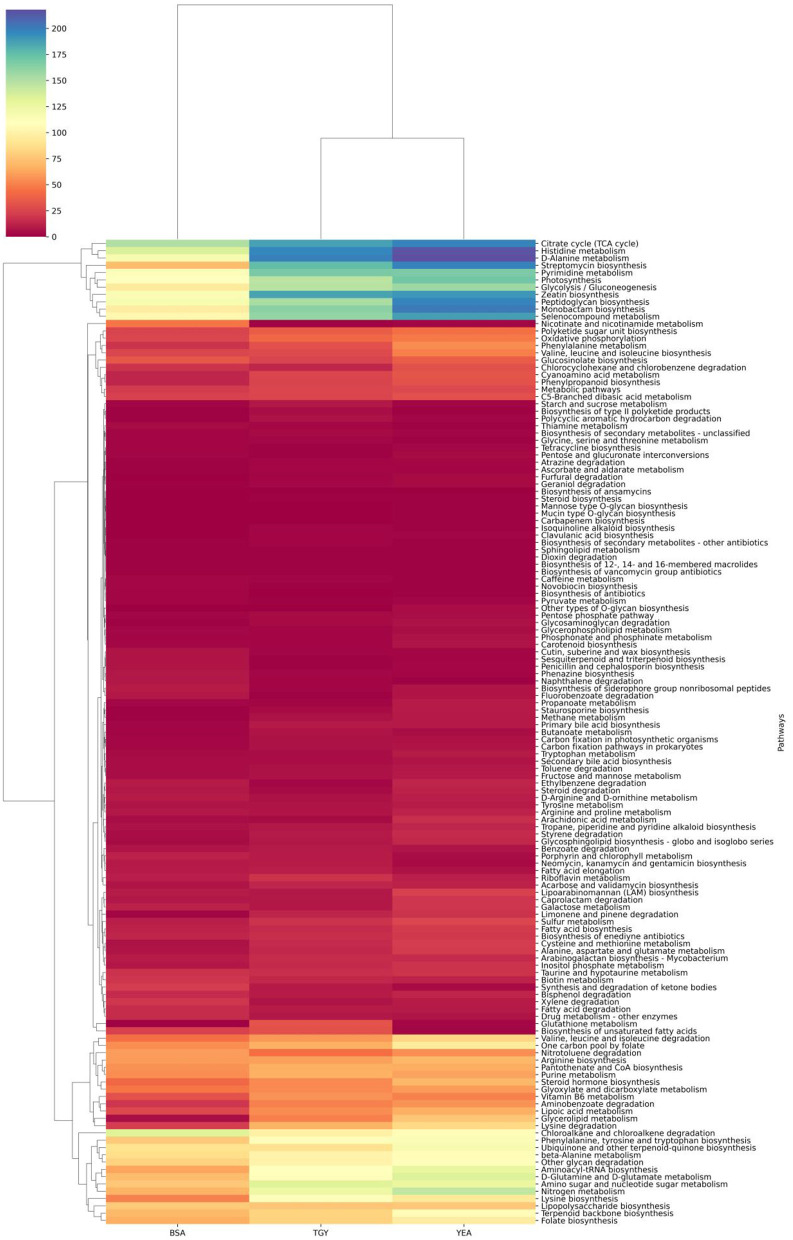
The figure presents a heatmap representing the KEGG pathway analysis for three locations along river Yamuna: BSA, TGY, and YEA. The analysis includes a hierarchical clustering of various metabolic pathways based on their relative abundance across the three locations. The color intensity in the heatmap reflects the abundance of each pathway, with warmer colors (red) indicating higher activity and cooler colors (blue) indicating lower levels. The pathways are organized along the *y*-axis, and the sampling locations (BSA, TGY, YEA) are displayed along the *x*-axis.

The KEGG pathway analysis provides insights into the functional metabolic profiles of microbial communities across all three locations along the river Yamuna (Figure 4). The heatmap generated from this analysis highlights the differential presence and abundance of metabolic pathways at these locations.

BSA showed a significant presence of diverse metabolic pathways, with many pathways showing high abundance. Notable pathways include the citric acid cycle (TCA cycle), glycolysis/gluconeogenesis, amino acid metabolism, and secondary metabolism. The data indicated that BSA supports a wide range of microbial processes, particularly those related to energy production (e.g., glycolysis and TCA cycle), nitrogen metabolism, and secondary metabolite biosynthesis. The TGY location showed a more specialized metabolic profile than BSA, with some pathways exhibiting lower abundance. Pathways such as aromatic compound degradation, aromatic amino acid metabolism, and secondary metabolism are more prominent. However, some primary metabolic pathways like glycolysis/gluconeogenesis and TCA cycle were still present but less abundant compared to BSA. YEA showed the lowest abundance of many metabolic pathways, with some pathways nearly absent. The heatmap indicated a relative scarcity of primary metabolic pathways such as glycolysis, TCA cycle, and amino acid metabolism. However, secondary metabolic pathways, including those related to biosynthesis of antibiotics and terpenoid backbone biosynthesis, appear more active in this region.

### Plastic degrading enzymes

**Figure 5:**
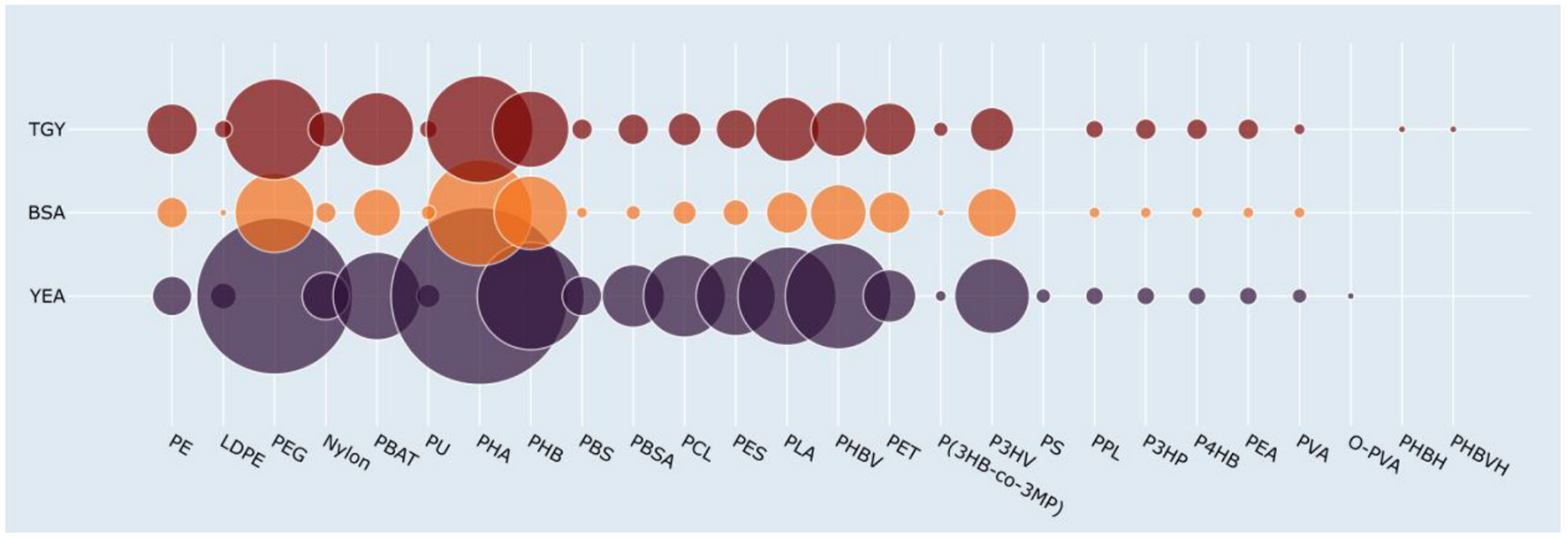
The metagenomic analysis of river Yamuna samples from three distinct locations (BSA, TGY, and YEA) highlights the variation in the distribution and abundance of plastic-degrading enzymes across a range of plastic types as identified in the PlasticDB database. Bubble size represents normalized abundance of enzyme-associated hits. It is important to note that the contribution of bacterial taxa to plastic degradation does not necessarily correlate with their overall abundance; some taxa with relatively lower population abundance may encode a higher number of plastic-degrading enzyme homologs, while more abundant taxa may contribute fewer such functions. This highlights the distinction between taxonomic abundance and functional potential within the microbial community.

The metagenomic analysis of the river Yamuna samples from the three different locations (BSA, TGY, and YEA) revealed notable differences in the distribution and prevalence of plastic-degrading enzymes across various types of plastics (Figure 5). The data, obtained from the PlasticDB database, showed a clear variation in microbial activity at the different sites, with YEA generally exhibiting the highest number of hits for most plastic types, suggesting that this location harbors a more diverse and active microbial community capable of degrading a wide variety of plastics. For instance, the distribution of plastic-degrading enzyme homologs revealed that the strongest signals were associated with polymers such as polyhydroxyalkanoates (PHA), polyhydroxybutyrate (PHB), and polyethylene glycol (PEG), which are relatively more biodegradable. In contrast, homologs associated with the degradation of more recalcitrant plastics, such as polyethylene (PE) and low-density polyethylene (LDPE), were detected at low abundance across all sites. These findings indicate that the microbial communities possess functional potential for degradation of biodegradable polymers, while the capacity for degradation of more persistent commodity plastics appears limited based on the current dataset.

In contrast, the presence of plastic-degrading enzymes for certain plastics, such as Polyurethane (PU) and Polyhydroxybutyrate-Hydroxyvalerate (PHBH) was comparatively low across all locations, with minimal hits recorded, particularly in BSA and TGY. Additionally, while plastics such as Polylactic Acid (PLA) and Polycaprolactone (PCL) showed moderate increases in hits at YEA, others, such as Polyethylene (PE) and Low-Density Polyethylene (LDPE) showed a more consistent but still relatively low level of degradation potential across all locations, especially in BSA. Polybutylene succinate adipate (PBSA) and Polyethylene terephthalate (PET) demonstrated a similar trend, with moderate increases in hits from BSA to YEA, especially for PBSA, which had a marked rise in microbial activity at YEA.

**Figure 6:**
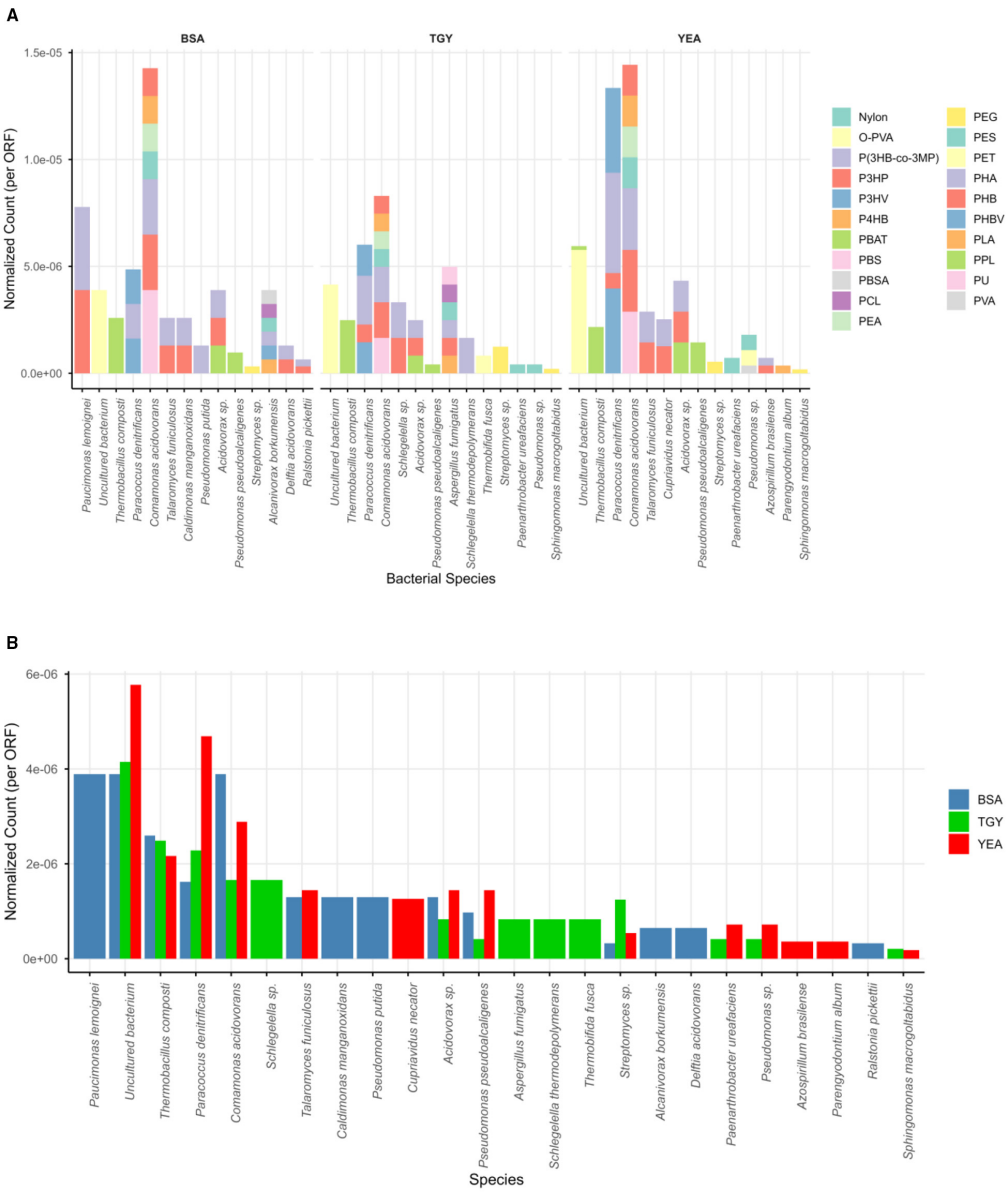
**(a)** The bar plot shows the diversity of bacterial species across three locations (BSA, TGY, and YEA) in the river Yamuna, with varying functional potential for plastic degradation. The normalized count of each bacterial species is represented along the *y*-axis, while the *x*-axis lists the species. Different colors represent various types that are degraded by bacteria, such as Nylon, Oxidized polyvinyl alcohol (O-PVA), P(3HB-co-3MP), P3HP, P3HV, and others. **(b)** The bar chart displays the distribution of plastic-degrading bacterial species across the three locations in the river Yamuna. The *y*-axis shows the count of species, and the *x*-axis lists the bacterial species identified at each location. All the plots were generated using R with the readxl, ggplot2, dplyr, and RColorBrewer libraries.

The distribution of plastic-degrading bacterial species across the three sampling locations (BSA, TGY, and YEA) was analyzed using normalized metrics to account for differences in sequencing depth and gene prediction. The normalization was based on total predicted ORFs. The bacterial species diversity across all three locations of the river Yamuna showed varying functional potential for plastic degradation (Figure 6a). An uncultured bacterium was the most abundant, degrading a wide range of plastics, including Nylon, Poly 3-hydroxypropionate (P3HP), and PHA, with the highest counts observed for these plastic types. This uncultured bacterium indicated the presence of taxa not well-represented in current reference databases. *Paracoccus denitrificans* followed closely, showing significant degradation of plastics such as polyethylene glycol (PEG), Polyvinyl alcohol (PVA) and Poly ε-caprolactone (PCL), though at lower frequencies than the uncultured bacterium. *Comamonas acidovorans* was another key player, predominantly degrading PET and PHA, with notable frequencies in PHA degradation. Other species, such as *Pseudomonas pseudoalcaligenes* and *Talaromyces funiculosus*, exhibited degradation potential for PCL and Poly butylene succinate (PBS), but in smaller proportions. *Sphingomonas macrogoltabidus* and *Ralstonia pickettii* showed lower counts, although they were capable of degrading plastics such as Poly 3-hydroxyvalerate (P3HV) and Polybutylene adipate terephthalate (PBAT).

The bar chart displays the distribution of plastic-degrading bacterial species across three locations along the river Yamuna (Figure 6b). The results showed significant variability in the presence and abundance of these species across locations. The uncultured bacterium was predominantly found in TGY and YEA, with high normalized counts based on total predicted ORFs in both, whereas it was less abundant in BSA. *Paucimonas lemnigri* was present only in BSA and TGY, being absent in YEA. *Paracoccus dentrificans* was present at all three locations, with the highest concentration in TGY. Similarly, *Thermobacillus composti* and *Schlegelella* sp. were observed primarily in BSA and YEA, with moderate counts in TGY. Other species, such as *Talaromyces funiculosus* and *Pseudomonas* sp. showed higher counts in BSA and YEA, TGY and YEA, respectively, while *Pseudomonas pseudoalcaligenes* was more prevalent in YEA. On the other hand, *Streptomyces* sp. and *Sphingomonas macrogolitabidus* were less abundant across all locations, indicating a lower occurrence of these species.

The contribution of bacterial taxa to plastic degradation varied across species and polymer types and does not directly correspond to their overall abundance. Certain taxa, such as *Pseudomonas* spp., were associated with a higher number of enzyme homologs and are therefore considered primary degraders capable of initiating polymer breakdown. In contrast, other taxa may function as secondary degraders or co-metabolizers, utilizing intermediate compounds generated during initial degradation. Additionally, some members of the microbial community may play stabilizing or supportive roles within the degradation consortium without directly contributing to polymer breakdown. This reflects the multi-stage nature of plastic and microplastic degradation, which involves initial depolymerization, intermediate metabolite processing, and eventual mineralization. Therefore, the dominance of specific taxa at a given site may indicate different stages or functional roles within the degradation process rather than direct degradation efficiency alone (Figure 5). These observations highlight the importance of interpreting plastic degradation as a community-driven process rather than attributing it to individual species.

### Xenobiotic degrading enzymes

**Figure 7:**
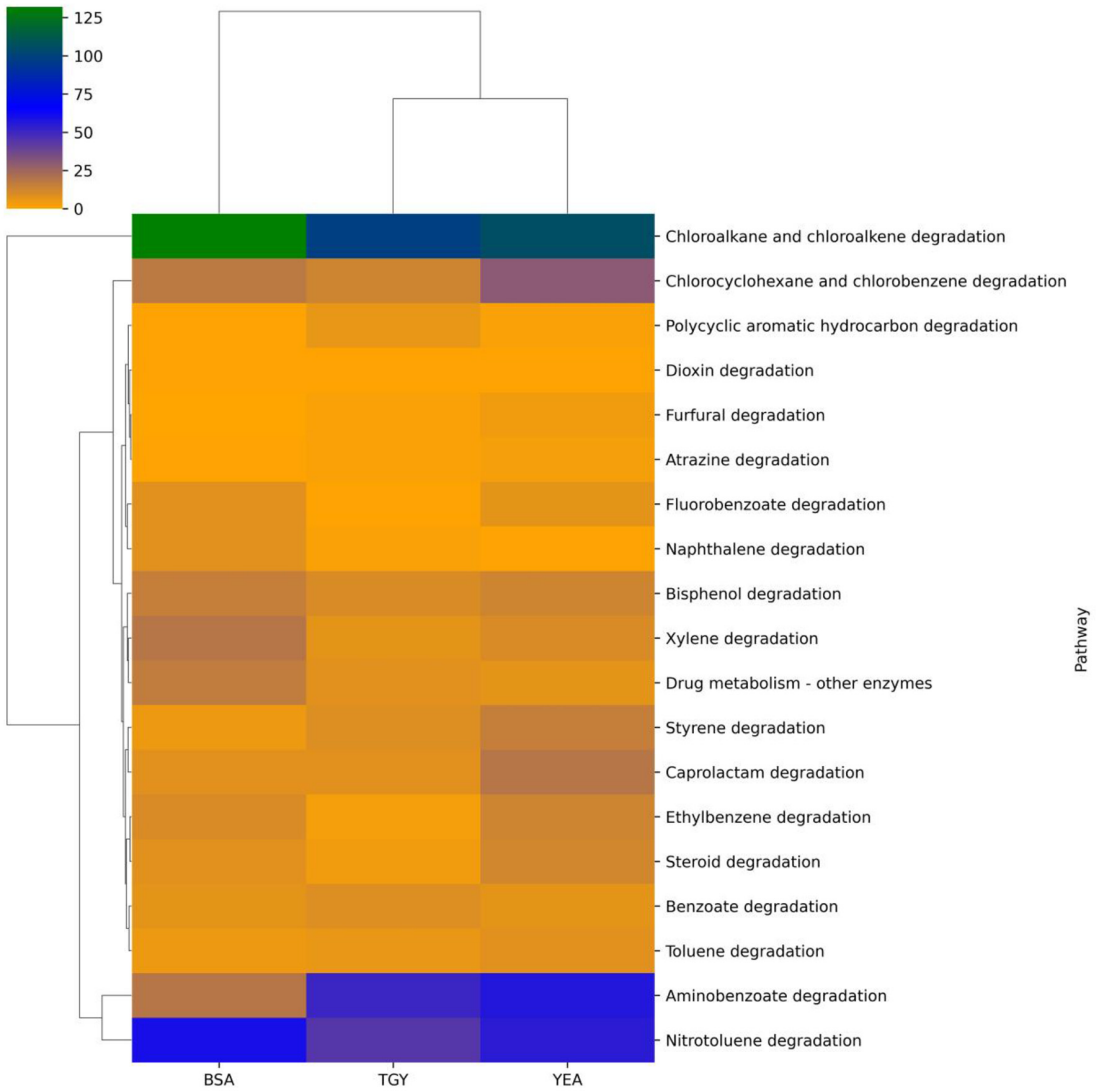
The heatmap represents the xenobiotic degradation pathway enzymes in three locations of the river Yamuna: BSA, TGY, and YEA. The color gradient indicates the relative abundance of bacterial species associated with the degradation of various xenobiotics, with darker shades representing higher abundance levels. The pathways depicted include chloralkane and chloralkene degradation, polycyclic aromatic hydrocarbon degradation, and other xenobiotic degradation pathways, such as toluene, xylene, and steroid degradation.

The metagenomic analysis of river Yamuna samples from three locations revealed varying levels of microbial involvement in xenobiotic degradation (Figure 7). These pathways, essential for degrading harmful synthetic compounds, exhibited distinct microbial activities across the locations. YEA exhibited the highest microbial activity in several degradation pathways, indicating a more diverse microbial community. For example, the chlorocyclohexane and chlorobenzene degradation pathway showed the highest number of hits at YEA, followed by BSA and TGY. Similarly, bisphenol degradation was most prominent at BSA, with YEA and TGY showing moderate activity. On the other hand, fluorobenzoate degradation was more prominent in BSA and YEA, with minimal hits in TGY.

Interestingly, pathways such as furfural degradation showed minimal microbial activity: BSA had no hits, while TGY and YEA showed slight activity (2 and 4 hits, respectively), indicating that furfural degradation is not a major microbial function in the river Yamuna. The degradation of aromatic compounds such as xylene and toluene was more active in BSA than in TGY and YEA, although YEA exhibited higher activity for toluene degradation. Polycyclic aromatic hydrocarbons (PAH), a class of persistent organic pollutants, were degraded more actively in TGY compared to BSA and YEA. The chloroalkane and chloroalkene degradation pathway stood out as the highest number of hits across all sites, particularly at BSA, followed by YEA and TGY. Similarly, aminobenzoate degradation was more prominent at YEA, followed by TGY and BSA. In contrast, naphthalene degradation showed minimal activity across all locations, particularly at YEA. Other degradation pathways, such as nitrotoluene and ethylbenzene, exhibited moderate activity at all sites, with YEA showing the highest microbial involvement for ethylbenzene degradation. Styrene degradation was also more active in YEA. Caprolactam degradation showed a significant increase in microbial activity from BSA to YEA, with TGY maintaining a consistent level of activity. Steroid degradation also followed a similar trend, with the highest number of hits at YEA, followed by BSA and TGY. Pathways like atrazine degradation, however, showed minimal microbial activity across all locations, with very few hits.

## Discussion

### Microbial diversity and ecological variability across the river Yamuna

The metagenomic analysis of microbial communities across the three distinct locations along the river Yamuna (BSA, TGY, and YEA) provided valuable insights into microbial diversity and composition. The taxonomic abundance analysis revealed clear differences in microbial community structure, indicating how species are distributed and how their abundance varies between locations. Such variations in river microbial communities are often influenced by ecological factors like geography, hydrology, and significant anthropogenic pressures, including pollution (Engloner et al., 2023; Regar et al., 2024; Cruz-Cano et al., 2025). The river Yamuna is recognized as a major polluted river in India, contaminated by sewage and other effluents (Mittal et al., 2019; Parida et al., 2022).

The BSA location exhibited a highly diverse and balanced microbial community, with a wide range of species contributing to the overall taxonomic composition. Notably, species such as *Pseudomonas junendi* and *Arcobacter venerupis* dominated the community, with other species, such as *Sulfurospirillum diekteriae, Hydrogenophaga taeniospiralis*, and *Pseudomonas aeruginosa*, contributing significantly to the microbial population. *Pseudomonas* species play key roles in carbon and nitrogen cycling by decomposition of various organic compounds, including pollutants (Engloner et al., 2023). The presence and abundance of *Arcobacter* are often associated with poor water quality in rivers and can be prevalent in sewage ecosystems (Godoy et al., 2020; Aishwarya et al., 2021). This suggests that BSA provides a more stable and diverse ecological niche in which a variety of microbial species can thrive. The relatively even distribution of species indicated that the local environment supports a rich microbial biodiversity, with different species coexisting with minimal competitive exclusion (Van Rossum et al., 2015). The diversity at BSA may reflect a balanced ecosystem with sufficient resources, proper nutrient availability, and stable environmental conditions. The presence of diverse species, especially those involved in primary and secondary metabolic processes such as nitrogen fixation, sulfur metabolism, and hydrocarbon degradation, suggests that BSA is likely an ecologically complex site where microbial communities can engage in a range of biochemical processes (Mittal et al., 2019; Yadav et al., 2021).

In contrast to BSA, the microbial diversity at TGY was less balanced, with some species showing higher dominance. The prominent species observed in TGY included *Acinetobacter* sp. *CS-2* and *Aliarcobacter butzleri*, indicating that these species may have competitive advantages or specialized metabolic capabilities in this location. Other species such as *Dechloromonas* sp. *HYN0024* and *Sideroxyarcus emersonii* showed consistent presence and higher fractional read abundances across multiple samples, suggesting that these species have adapted to the specific environmental conditions at TGY. *Acinetobacter* species are frequently reported in polluted water and sewage sites, where they can be among the most abundant genera and harbor antibiotic resistance genes (Mittal et al., 2019; Ott et al., 2021; Lenart-Boroń et al., 2022). Furthermore, *Dechloromonas* species are recognized for their efficiency in degrading chlorinated or aromatic micropollutants (Engloner et al., 2023), and species like *Dechloromonas aromatica* have been identified as highly dominant in polluted stretches of river Yamuna, signifying the impact of pollution (Parida et al., 2022). The microbial community at TGY appears to be more specialized, with certain species potentially outcompeting others. This could be attributed to specific environmental factors such as localized nutrient availability, water quality, or other ecological pressures that favor the growth of certain microbial taxa over others (Zeglin, 2015; Chen et al., 2021). The presence of *Acinetobacter* species, known for their resistance to a variety of environmental stresses, could suggest that TGY is subject to conditions that favor such resilient organisms, potentially including high concentrations of pollutants or other anthropogenic influences (Kisková et al., 2023; Wang et al., 2024).

The microbial community at YEA was notably distinct from both BSA and TGY. YEA exhibited lower species abundance, with many species showing minimal fractional read abundance. Key species observed in YEA included *Aliarcobacter butzleri, Acinetobacter baumannii*, and *Dechloromonas denitrificans*, but these species had lower relative abundance compared to the other locations. Interestingly, *Pseudomonas aeruginosa* was more prominent in the YEA area, suggesting that this species might be adapted to specific conditions in this location. *Pseudomonas aeruginosa* is considered an opportunistic pathogen, and its persistence in river water can be influenced by various abiotic and biotic factors (Engloner et al., 2023; Gao et al., 2024). Reduced microbial diversity in YEA may indicate environmental stressors or ecological imbalances that limit microbial growth (Zeglin, 2015; Piergiacomo et al., 2022). This could be related to factors such as pollution, altered water chemistry, or other anthropogenic activities impacting microbial populations (Regar et al., 2024; Cruz-Cano et al., 2025). The lower abundance of key species at YEA may suggest that the environment is less conducive to supporting a diverse community of microorganisms. The presence of *Pseudomonas aeruginosa*, a known opportunistic pathogen, further supports the hypothesis that the YEA environment may be experiencing ecological disturbances that favor particular stress-tolerant or pathogenic species (Ott et al., 2021; Gao et al., 2024).

Across the three sites, there is clear variation in microbial community composition: BSA exhibits higher diversity and evenness, TGY hosts a more specialized community with dominant species, and YEA shows reduced diversity, potentially due to environmental stressors. These findings highlight the dynamic nature of microbial communities in river ecosystems and suggest that environmental factors, including water quality, nutrient availability, and pollution levels, play a crucial role in shaping microbial diversity (Woodward et al., 2012; Chen et al., 2021; Engloner et al., 2023). The results also underscore the importance of continuous monitoring of microbial communities to assess the health of aquatic ecosystems. The presence of stress-tolerant or pathogenic species in YEA may signal potential environmental degradation or ecological imbalances that could have broader implications for the river's health and biodiversity. Further research is needed to identify the specific environmental factors driving these observed patterns and to investigate the functional roles of the dominant microbial species in each location.

The observed spatial variation in microbial composition and functional potential along the river Yamuna is consistent with patterns reported in other South Asian river systems, where microbial communities are strongly shaped by anthropogenic pressures, hydrological variability, and physicochemical gradients. Recent studies on the Ganges–Yamuna basin highlight that pollution inputs, seasonal flow, and nutrient dynamics collectively drive shifts in microbial diversity and metabolic functions, reinforcing the role of river microbiomes as indicators of environmental stress and ecosystem health (Chaturvedi et al., 2024).

### BSA dominated energy metabolism in metabolic pathway diversity

The KEGG pathway analysis provided valuable insights into microbial metabolic functions along the river Yamuna by examining the abundance of metabolic pathways across the three distinct locations. Metagenomic studies are increasingly employed to understand the functional potential of microbial communities in river ecosystems, which are significantly influenced by environmental and anthropogenic factors (Yadav and Dharne, 2024; Borton et al., 2025). The taxonomic abundance analysis revealed clear differences in microbial community structure, indicating how species are distributed and how their abundance varies between locations, a pattern frequently observed in river microbiomes under varying environmental pressures (Regar et al., 2024).

BSA demonstrated a highly diverse and abundant presence of metabolic pathways. The heatmap highlights the dominance of energy-generating pathways such as the citric acid cycle (TCA cycle) and glycolysis/gluconeogenesis, which are key to microbial energy production (Zhang et al., 2024). These pathways suggest that the microbial communities in BSA are highly active in energy metabolism, representing core metabolic functions essential for cellular and ecosystem homeostasis (Santos-Júnior et al., 2020). Additionally, amino acid metabolism and secondary metabolism pathways were prominent, indicating that microbes in this area are involved in nitrogen cycling and the synthesis of bioactive compounds (Mittal et al., 2019). The TCA cycle, a central metabolic pathway for carbon metabolism, was notably abundant, underscoring the region's capability to support a broad array of microbial activities essential for growth and energy production, typically observed in less impacted aquatic environments (Beale et al., 2017; Santos-Júnior et al., 2020). The relatively even distribution of species indicated that the local environment supports a rich microbial biodiversity, with different species coexisting with minimal competitive exclusion, often reflective of balanced ecosystems (Van Rossum et al., 2015). The diversity at BSA may reflect a balanced ecosystem with sufficient resources, proper nutrient availability, and stable environmental conditions. The presence of diverse species, especially those involved in primary and secondary metabolic processes, suggests that BSA is likely an ecologically complex site, where microbial communities can engage in a range of biogeochemical processes, including nitrogen fixation cycling, sulfur metabolism, and hydrocarbon degradation (Mittal et al., 2019; Parida et al., 2022).

In contrast, TGY displayed a more specialized metabolic profile. While pathways such as glycolysis/gluconeogenesis and the TCA cycle were still present, their abundance was significantly lower than that observed in BSA. Instead, pathways related to the degradation of aromatic compounds and the metabolism of aromatic amino acids were more pronounced (Mittal et al., 2019; Yadav et al., 2021). This suggests that TGY supports a microbial community capable of utilizing specific organic compounds, including those derived from aromatic compounds, which is often indicative of environmental conditions with xenobiotic pollutant input (Breton-Deval et al., 2020; Zhang et al., 2020). Such adaptations highlight functional specialization in perturbed environments (Breton-Deval et al., 2020). Secondary metabolism also played a notable role, with pathways associated with the biosynthesis of specialized metabolites, such as antibiotics, being more active in TGY. The increased prevalence of antibiotic biosynthesis in river environments is often linked to anthropogenic contamination, particularly from wastewater effluents (Godoy et al., 2020; Lee et al., 2023).

YEA exhibited a contrasting pattern, with many metabolic pathways showing low abundance. Primary metabolic pathways such as glycolysis, the TCA cycle, and amino acid metabolism were notably scarce. This indicated that microbial communities in YEA may be less active in core energy production processes compared to the other locations. However, secondary metabolic pathways, including antibiotic biosynthesis and terpenoid backbone biosynthesis, were more prominent. This suggests that while the microbial community in YEA may not be heavily involved in primary energy production, it actively synthesizes secondary metabolites that could serve ecological functions such as defense or interactions with other microorganisms. This suggests that while the microbial community in YEA may not be heavily involved in primary energy production, it actively synthesizes secondary metabolites that could serve ecological functions such as defense or interactions with other microorganisms in response to environmental challenges (Byl et al., 2023). The reduced microbial diversity in YEA could indicate environmental stressors or ecological imbalances that limit microbial growth (Li et al., 2018; Mansfeldt et al., 2020). The lower abundance of key species at YEA may suggest that the environment is less conducive to supporting a diverse community of microorganisms. The presence of Pseudomonas aeruginosa, a known opportunistic pathogen, further supports the hypothesis that the YEA environment may be experiencing ecological disturbances that favor particular stress-tolerant or pathogenic species, a characteristic often observed in polluted river segments (Li et al., 2022; Xiao et al., 2023).

### Highest functional potential for plastic degradation at YEA

The results of the metagenomic analysis of plastic-degrading enzymes and bacteria across three locations along the river Yamuna showed microbial diversity and plastic degradation potential at these sites. Metagenomics offers a powerful approach to identify novel microbial communities and their functional genetics related to plastic degradation (Wani et al., 2023), particularly in riverine systems, which have shown substantial enrichment in plastic degraders (Ridley Jr et al., 2024). The findings highlight significant variations in microbial activity across locations, particularly in the degradation of different plastic types, offering crucial information regarding microbial communities capable of mitigating plastic pollution in this river system (Saleem et al., 2023).

The analysis of plastic-degrading enzymes from the PlasticDB database revealed notable differences in microbial activity at these sites. YEA exhibited the highest number of enzyme hits across most plastic types, suggesting a more active and diverse microbial community capable of degrading a wider range of plastics. For example, Polyhydroxyalkanoates (PHA) displayed a marked increase in enzyme hits from BSA to YEA. This increase indicates that YEA harbors a greater microbial capacity for PHA degradation, likely due to specialized microbial populations adapted to this bioplastic (Paloyan et al., 2025). Studies have shown that PHA degradation rates are influenced by environmental factors such as temperature and water composition (Volova et al., 2007; Voinova et al., 2008). Similarly, Polyhydroxybutyrate (PHB) was most abundant at YEA, followed by BSA and TGY. This trend suggests that the microbial communities at YEA are particularly efficient in degrading PHB, a biodegradable plastic commonly used in industrial applications. The increase in degradation potential of PHB at YEA could be linked to local environmental conditions and microbial adaptation to this plastic type. Other plastics, such as Polyethylene glycol, also showed a significant rise in microbial activity at YEA, further supporting the idea that this site hosts a more diverse microbial population with enhanced plastic-degrading capabilities, consistent with observations of microbial PEG degradation in various environments (Kawai, 1997). In contrast, certain plastics, such as Polyurethane and Polyhydroxybutyrate-Hydroxyvalerate, displayed low degradation potential across all three locations, with minimal enzyme hits recorded. This suggests that the microbial communities at BSA, TGY, and YEA may be less adapted to degrade these specific plastics, highlighting the need for further exploration of microbial strains capable of degrading a broader spectrum of plastic types in the river Yamuna (Khatua et al., 2024).

The observed enrichment of enzyme homologs associated with PHA and PHB degradation may be linked to localized anthropogenic inputs along the river Yamuna. These polymers are biodegradable and are commonly associated with organic-rich waste streams, including domestic sewage, food waste, and biodegradable plastic materials. In the present study, sites such as TGY, which are influenced by dense urban activity and sewage discharge, likely receive higher inputs of such biodegradable substrates, supporting microbial communities capable of utilizing PHA- and PHB-like compounds. Similarly, the relatively elevated signals observed at YEA may reflect cumulative downstream inputs of organic waste and biodegradable materials transported from upstream urban centers. Industrial discharges, particularly from small-scale processing units and plastic-related activities in the Agra region, may also contribute to the presence of polymer-associated substrates. These findings suggest that the detected functional potential for PHA/PHB degradation is consistent with the environmental context of the sampling sites; however, the results should be interpreted as indicative of substrate availability and microbial adaptation rather than direct evidence of active polymer degradation. This interpretation is supported by elevated organic carbon and BOD levels observed at these sites, indicating increased organic loading.

Plastics like Polylactic Acid (PLA) and Polycaprolactone (PCL) showed moderate increases in microbial activity at YEA, indicating some capacity for degradation, although this was less pronounced than that for PHA and PHB. Biodegradation of these bioplastics can occur under aqueous conditions, and the process is influenced by the polymer's chemical structure and environmental factors (Kikuchi et al., 2024; García-Depraect et al., 2022). On the other hand, Polyethylene (PE) and Low-Density Polyethylene (LDPE) exhibited relatively low degradation potential across all the sites, especially at BSA, where the microbial activity was minimal. This could be due to the recalcitrant nature of PE and LDPE, which are more challenging for microbes to break down (Muangchinda and Pinyakong, 2024). Despite this, certain bacterial genera such as Pseudomonas and Bacillus have been identified as capable of degrading PE and LDPE (Lee et al., 2020; Agarwal et al., 2024). Plastics such as Polybutylene succinate adipate (PBSA) and Polyethylene terephthalate (PET) also showed moderate increases in microbial activity from BSA to YEA, with PBSA showing a more significant rise, particularly at YEA. This suggests that the microbial populations at YEA may possess the specialized enzymes necessary for the degradation of PBSA, which is increasingly being used as an alternative biodegradable plastic in packaging (Guliyev et al., 2022; Yokoyama et al., 2023).

Analysis of bacterial species diversity across the three sampling locations revealed a varying functional potential for plastic degradation. The most prominent species in microbial communities was an uncultured bacterium that exhibited high degradation capacities for plastics such as Nylon, P3HP, and PHA. This bacterium was particularly abundant at TGY and YEA, with its counts significantly higher than at BSA. This indicates that this uncultured bacterium plays a crucial role in plastic degradation at these locations and is likely a key player in mitigating plastic pollution in the river. The importance of uncultivated microbial species in various environmental processes, including plastic degradation, is increasingly recognized through metagenomic studies (Herrera et al., 2023).

Other critical bacterial species involved in plastic degradation included *Paracoccus denitrificans*, which was present at all three locations, with the highest concentrations at TGY. *Paracoccus denitrificans* associated ORFs exhibited degradation potential for plastics such as PEG, PVA, and PCL degrading homologs, suggesting its versatility in degrading diverse plastics. *Paracoccus* species are known for their role in biodegradation processes (Satta et al., 2024). *Comamonas acidovorans*, another significant species, carry linked ORFs for the degradation of PET and PHA. It showed notable frequencies of PHA degradation across all three sites, although it was more prevalent at TGY and YEA. This indicates that *Comamonas acidovorans* may be particularly adapted to environments where PHA is more prevalent, further emphasizing the site-specific nature of microbial degradation processes. Other bacterial species, such as *Pseudomonas pseudoalcaligenes* and *Talaromyces funiculosus*, showed lower counts but were still significant contributors to plastic degradation at YEA. *Pseudomonas pseudoalcaligenes* associated ORFs was predominantly found in TGY and exhibited degradation potential for PCL and PBS. *Talaromyces funiculosus*, another key player, was more prevalent at YEA, where it contributed to the degradation of PCL and PBS. Fungi are well-documented for their ability to degrade various plastics, including PCL (Kim et al., 2022; Khatua et al., 2024). This further reinforces the idea that YEA appears to harbor a microbial community with higher relative abundance of plastic-degrading enzyme homologs, which may reflect localized accumulation of substrates and environmental selection rather than direct evidence of source-specific enrichment. In contrast, species like *Sphingomonas macrogolitabidus* and *Ralstonia pickettii* associated ORFs were less abundant across all locations, suggesting that their functional potential for plastic degradation in the river Yamuna is limited. These species showed a lower occurrence, indicating that their roles in plastic biodegradation might be less significant compared to other, more prevalent species.

The site-specific variation in microbial diversity and plastic degradation potential highlights the influence of local environmental factors on microbial communities. Abiotic factors such as temperature, pH, and plastic waste availability are crucial in influencing microbial activity and plastic degradation (Salinas et al., 2023; Ventura et al., 2024). At TGY and YEA, the higher abundance of plastic-degrading species such as the uncultured bacterium, *Paracoccus dentrificans*, and *Talaromyces funiculosus* suggests that these sites provide a more conducive environment for microbial growth and plastic degradation. This could be attributed to factors such as water temperature, pH, and plastic waste availability, which may vary across sites. BSA, on the other hand, exhibited a more limited diversity of plastic-degrading bacteria, with species such as *Thermobacillus composti* and *Schlegelella* sp. being the most prominent. These species were observed primarily at BSA and YEA, indicating that BSA might have had a less diverse microbial population capable of degrading a wide range of plastics. This site-specific microbial distribution further emphasizes the importance of environmental factors in shaping microbial communities and their plastic-degrading capacities (Niu et al., 2025).

The metagenomic analysis revealed the presence of homologs to enzymes associated with plastic degradation; however, this functional signal was predominantly driven by polymers such as PHA, PHB, and PEG, which are known to be more readily biodegradable. In contrast, enzyme homologs linked to the degradation of more recalcitrant plastics, including polyethylene (PE) and low-density polyethylene (LDPE), were comparatively scarce. This distinction is important, as biodegradable polymers differ substantially from conventional commodity plastics in terms of environmental persistence and degradation mechanisms. Therefore, the observed patterns should be interpreted as evidence of functional potential primarily for biodegradable polymer turnover rather than a broad capacity to degrade persistent plastic pollutants. Furthermore, as these results are based on homology-driven annotation, they reflect potential metabolic capability rather than confirmed *in situ* degradation activity. Additional functional validation, such as enzyme assays or transcriptomic analysis, would be required to confirm active biodegradation processes.

A key strength of the present study is the integration of functional annotation and taxonomic classification at the ORF level, enabling direct linkage of plastic-degrading enzyme homologs to specific taxa within the metagenomic datasets. This approach improves upon database-only inference by providing evidence that these functional genes are encoded by taxa present in the sampled environments. Nevertheless, it is important to note that homology-based annotation indicates functional potential rather than confirmed enzymatic activity. While the presence of these genes suggests the capability for plastic degradation, further validation through transcriptomic, proteomic, or culture-based assays would be required to confirm active biodegradation under *in situ* conditions.

### Chlorocyclohexane and chlorobenzene degradation dominate at YEA

The xenobiotic degradation pathway analysis identified key microbial activities involved in degradation of xenobiotics across three distinct sampling locations in the river Yamuna. This metagenomic analysis revealed varying levels of microbial involvement in the breakdown of these compounds, which is critical for understanding microbial dynamics in polluted river environments (Mittal et al., 2019; Breton-Deval et al., 2020). Rapid industrialization significantly contributes to the accumulation of xenobiotic pollutants in rivers, necessitating an understanding of microbial functional potential for bioremediation (Yadav et al., 2021; Yadav and Dharne, 2024).

The diversity of microbial activity was most pronounced at YEA, where several degradation pathways exhibited high levels of activity. This site exhibited the highest microbial activity for the chlorocyclohexane and chlorobenzene degradation pathways, suggesting a diverse microbial community capable of degrading complex organochlorine compounds (Dechesne et al., 2018; Zhang et al., 2019). This was followed by BSA and TGY, where the microbial activity was more moderate, particularly for these chlorinated compounds. This indicates that while all sites possess microorganisms capable of degrading chlorocyclohexane and chlorobenzene, the microbial community at YEA might be more specialized or abundant in these pathways, which is crucial given that chlorobenzene is a common industrial solvent and priority pollutant (Zhang et al., 2019; Pan et al., 2025).

In terms of bisphenol degradation, BSA exhibited the highest microbial activity, which was noteworthy considering bisphenol is a common contaminant in water bodies. Bisphenol A is a common contaminant in water bodies, often entering through wastewater treatment plant effluents (Noszczyńska et al., 2021). Microbial degradation is a known mechanism for its removal from environments such as rivers (Zhou et al., 2020; Noszczyńska et al., 2021). YEA and TGY displayed more moderate activity in bisphenol degradation, suggesting possible site-specific variations in microbial composition and function (Noszczyńska et al., 2023). The ability of bacteria such as *Pseudomonas* and *Sphingomonas* to degrade BPA has been well-documented (Noszczyńska et al., 2021; Han et al., 2023; Jahanshahi et al., 2023). Similarly, fluorobenzoate degradation was prominent in BSA and YEA, indicating that these locations harbor microbial communities capable of degrading such aromatic pollutants, which are known to be persistent in aquatic environments.

One of the key findings from the analysis was the relatively low microbial activity for furfural degradation, with BSA showing no hits and YEA showing minimal hits. Furfural is a toxic compound typically found in industrial wastewater and is considered refractory in aqueous media (Rahmani et al., 2020; Jabbar et al., 2023). The lack of significant degradation activity may reflect the absence of specific microbial taxa capable of processing this compound, or potentially the lower concentration of furfural in the river at the time of sampling. Despite their toxicity, certain microorganisms, such as *Acinetobacter baylyi*, are known to metabolize furan aldehydes (Liu et al., 2024), and bacterial consortia can be adapted for their biodegradation (Farias et al., 2025). The degradation of aromatic hydrocarbons, such as xylene and toluene, was more pronounced in BSA, whereas YEA showed higher activity toward toluene degradation. Metagenomic studies of the river Yamuna have identified complete degradation pathways for aromatic compounds, including toluene and xylene (Mittal et al., 2019). This suggests that BSA hosts a more active microbial community for certain aromatic pollutants, whereas YEA may have more diverse pathways for other aromatic hydrocarbons, such as toluene. Biodegradation of these compounds is a major process for their removal in aquatic environments (Søndergaard et al., 2017; Chen et al., 2022).

Another interesting observation was the degradation of polycyclic aromatic hydrocarbons (PAHs), which was more active at TGY than at the other locations. PAHs are persistent organic pollutants, and their degradation can be a crucial ecological function for mitigating the long-term environmental impact of industrial pollution (Premnath et al., 2021). Microbial degradation is a major process for PAH removal in sediments and water (Yan et al., 2017; Premnath et al., 2021). This result indicates that TGY harbors microorganisms that are particularly efficient in PAH degradation, highlighting the specialized metabolic capabilities at this location, which are critical for mitigating the long-term environmental impact of industrial pollution (Yuan et al., 2001). The chloroalkane and chloroalkene degradation pathways exhibited the highest overall microbial activity across all three locations, especially at BSA. This is a critical finding, as these pathways are involved in the degradation of common pollutants such as chlorinated solvents, which are widespread in industrial wastewater (Tiehm and Schmidt, 2011; Ottosen et al., 2020). Degradation of chloroethenes (a type of chloroalkene) can occur under both anaerobic and aerobic conditions in contaminated groundwater and stream water (Tiehm and Schmidt, 2011; Rønde, 2019; Weatherill et al., 2019).

Aminobenzoate degradation was more prominent at YEA, followed by TGY and BSA, suggesting that the microbial communities at YEA might be enriched for microorganisms capable of degrading this compound. Catabolic genes for aminobenzoate have been detected in polluted urban river systems (Yadav et al., 2021). Similarly, degradation of nitrotoluene and ethylbenzene was moderate across all sites, but YEA exhibited the highest activity for ethylbenzene degradation. This suggests that YEA could be more effective at degrading some of the more complex aromatic compounds, including ethylbenzene, a compound commonly found in industrial waste (Søndergaard et al., 2017; Chen et al., 2022). Caprolactam degradation showed an increasing trend from BSA to YEA, which might indicate a gradual shift in microbial communities that are capable of processing this compound, potentially due to changes in pollution sources or microbial adaptation over time. Steroid degradation also showed an increasing trend from BSA to YEA, which further emphasizes the shift in microbial capabilities across the sampling sites. These findings are significant because steroid degradation can impact the removal of hormones and other biologically active compounds from water, which could have downstream ecological implications.

In addition to these, pathways such as atrazine degradation, a widely used herbicide, showed minimal microbial activity at all locations. Atrazine is a well-known herbicide whose residue seriously pollutes the environment (Bao et al., 2024). While microbial degradation of atrazine can occur, its efficiency varies (Satsuma, 2009; Zhao et al., 2023). This suggests that while other compounds might be targeted by the microbial communities, there may be a lack of specific microbial taxa capable of degrading atrazine efficiently in the river Yamuna (Hurtado et al., 2016; De Rosa et al., 2024). Adsorption to sediment and microbial N-dealkylation are key processes in its environmental fate (Triassi et al., 2022; De Rosa et al., 2024).

A key limitation of the present study is that sampling was conducted at a single time point and therefore does not capture temporal variability associated with seasonal dynamics. River systems such as the Yamuna are strongly influenced by seasonal changes, particularly monsoon-driven runoff, which can increase pollutant loading, sediment resuspension, and nutrient influx, as well as dry-season conditions that may concentrate contaminants due to reduced flow. These factors can significantly influence microbial community composition and functional gene abundance. Accordingly, the results presented here represent a snapshot of the microbial and functional profile under specific environmental conditions and may not fully reflect seasonal variability. Future studies incorporating multi-seasonal sampling and longitudinal monitoring will be essential to better understand temporal dynamics and their influence on microbial-mediated biodegradation potential.

Another key limitation of the present study is the absence of explicit hydrological characterization, including parameters such as flow velocity, discharge, sedimentation rate, and resuspension dynamics. These factors strongly influence sediment structure, pollutant transport, and microbial community assembly in riverine systems. As a result, the observed distribution of plastic- and xenobiotic-degrading microbial communities likely reflects zones of pollutant accumulation and local ecological conditions rather than direct source attribution. Furthermore, sediment layers sampled at 10–20 cm depth may not represent fully stabilized microbial communities but could instead comprise transitional assemblages influenced by ongoing deposition and resuspension processes. Consequently, caution should be exercised in interpreting these findings as definitive indicators of pollution sources or long-term microbial equilibrium.

In addition to metagenomic approaches, emerging technologies such as hyperspectral remote sensing provide complementary tools for large-scale monitoring of water quality. Hyperspectral sensors can detect variations in water constituents such as dissolved organic matter, suspended solids, and chlorophyll through spectral signatures, enabling real-time and spatially extensive assessment of pollution dynamics (Srivastava et al., 2025). Integration of such remote sensing approaches with metagenomic data can enhance environmental monitoring frameworks by linking microbial functional potential with physicochemical changes at ecosystem scales. Such integrated approaches are also aligned with global sustainability priorities, particularly Sustainable Development Goals (SDG 6: Clean Water and Sanitation and SDG 12: Responsible Consumption and Production), by supporting improved water quality assessment, pollution mitigation, and sustainable resource management (Chaturvedi et al., 2025a,b).

## Conclusions

This study highlights site-specific patterns of microbial diversity and bioremediation potential across three locations along the river Yamuna. BSA exhibited the highest taxonomic diversity and enrichment of energy metabolism pathways, indicating a metabolically robust microbial community. In contrast, YEA demonstrated a pronounced capacity for xenobiotic degradation, particularly targeting complex organochlorine compounds such as chlorocyclohexane and chlorobenzene. Distinct site-specific degradation profiles were evident: BSA was predominantly associated with bisphenol degradation, whereas TGY showed enhanced potential for polycyclic aromatic hydrocarbon (PAH) catabolism. These observations underscore the role of environmental selection in shaping microbial functional guilds involved in pollutant degradation and emphasize the importance of targeted monitoring and functional validation of key taxa to support effective riverine bioremediation strategies. In addition, sediment microbial communities were found to harbor genes encoding plastic-degrading enzymes, particularly those involved in the breakdown of biodegradable polymers such as polyhydroxyalkanoates (PHA), polyhydroxybutyrate (PHB), and polyethylene glycol (PEG). However, the relatively low abundance of homologs associated with the degradation of more recalcitrant plastics, including polyethylene (PE) and low-density polyethylene (LDPE), suggests a limited inherent capacity for degrading persistent plastic pollutants. Overall, these findings highlight the functional potential of sediment-associated microbial communities for polymer degradation, while emphasizing the need for further experimental validation to establish their practical applicability in plastic bioremediation. The metagenomic insights generated in this study also have broader relevance for global sustainability frameworks, particularly the Sustainable Development Goals, including clean water and sanitation (SDG 6) and responsible consumption and production (SDG 12). A deeper understanding of microbial-mediated degradation pathways can contribute to improved water resource management and support strategies aimed at pollution mitigation and enhanced resilience of freshwater ecosystems. However, these findings should be interpreted in the context of hydrological influences on sediment dynamics, as the absence of flow-related measurements limits the ability to distinguish between pollutant accumulation zones and true source contributions.

## Data Availability

The datasets presented in this study can be found in online repositories. The names of the repository/repositories and accession number(s) can be found in the article/Supplementary material.
