# Supplementary material for: Metagenomic insights into microbial diversity, xenobiotic and plastic-degrading enzymes in sediments of river Yamuna at Agra
Source: Front Microbiol. 2026 Jun 3;17:1828736. doi: 10.3389/fmicb.2026.1828736 (PMC13272299; doi:10.3389/fmicb.2026.1828736)
Supplement: Supplementary file 1 [file Table_1.DOCX]

**Table S1.** Physicochemical parameters and heavy metals of water at different sampling sites

| **Parameters** | **BSA** | **TGY** | **YEA** |
| --- | --- | --- | --- |
| DO (ppm) | 4.8 | 4.6 | 2.8 |
| pH | 7.96 | 7.1 | 7.4 |
| TDS (ppm) | 1606 | 782 | 754 |
| ORP (mV) | 122.6 | 238.4 | 29.9 |
| Specific conductivity (µS/cm) | 1050 | 950 | 859 |
| Salinity (psu) | 0.84 | 0.96 | 0.86 |
| BOD (ppm) | 23.9 | 6.7 | 4.2 |
| COD (ppm) | 13.2 | 32.9 | 18.9 |
| Calcium (ppm) | 14.1 | 15.6 | 17.2 |
| Magnesium (ppm) | 12.8 | 18.4 | 19.6 |
| Potassium (ppm) | 3.8 | 4.6 | 5.8 |
| Sodium (ppm) | 19.8 | 21.4 | 22.1 |
| Ammonium-N (ppm) | 0.31 | 0.35 | 0.41 |
| Nitrate-N (ppm) | 0.12 | 0.1 | 0.09 |
| Total Phosphate (ppm) | 0.23 | 0.22 | 0.23 |
| Sulphate (ppm) | 17.5 | 20 | 19.1 |
| Manganese (µg/L) | 383.0 | 210.1 | 638.0 |
| Iron (µg/L) | 94.8 | 239.3 | 1444.0 |
| Copper (µg/L) | 43.3 | 274.0 | 113.0 |
| Zinc (µg/L) | 58.1 | 892.6 | 288.5 |
| Cadmium (µg/L) | 0.0 | 121.2 | 5.1 |
| Lead (µg/L) | 1.5 | 3.0 | 9.0 |
| Chromium (µg/L) | 178.7 | 29.5 | 65.1 |


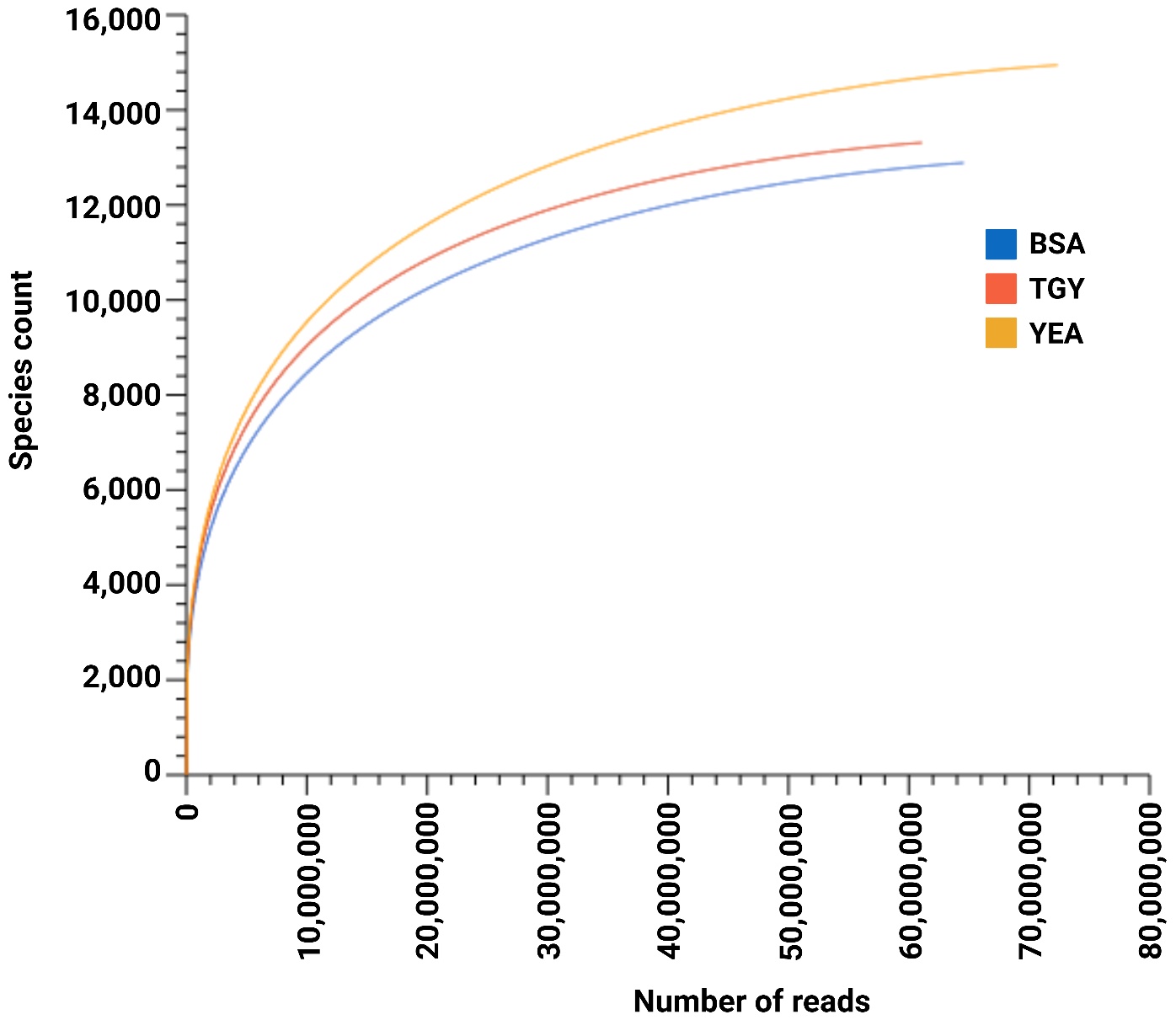


**Fig. S1** A rarefaction curve illustrates the species richness of individual metagenome samples identified in shotgun metagenome datasets, with each curve corresponding to a specific sample site. The x-axis denotes the number of reads in millions, while the y-axis represents species richness.
